# Total shoulder arthroplasty for glenohumeral osteoarthritis leads to better outcomes than hemiarthroplasty at a minimum 5 years: an intraoperative randomization-controlled trial of 79 patients

**DOI:** 10.2340/17453674.2025.44946

**Published:** 2025-12-11

**Authors:** Norbert SÜDKAMP, Martin JAEGER, Lars ADOLFSSON, Thomas BERNDT, Michael BLAUTH, Alexander JOERIS, Simon LAMBERT

**Affiliations:** 1 Department of Orthopedics and Traumatology, University Medical Center Freiburg, Freiburg, Germany; 2 Department of Orthopaedics, Linköping University, Linköping, Sweden; 3 Clinic for Orthopedics, Traumatology and Sports Medicine, Hannover Regional Clinic, Laatzen, Germany; 4 Department of Trauma Surgery and Sports Medicine, Medical University Innsbruck, Innsbruck, Austria; 5 AO Innovation Translation Center, Medical Scientific Affairs, AO Foundation, Davos, Switzerland; 6 The Princess Grace Hospital, London, UK

## Abstract

**Background and purpose:**

It is unclear whether total shoulder arthroplasty (TSA) results in better outcomes in patients with degenerative shoulder diseases compared with hemiarthroplasty (HA). This randomized controlled trial (NCT01288066) is an international, multicenter study with the primary aim to assess pain and shoulder joint function at 5-year follow-up in adults surgically treated with TSA or HA.

**Methods:**

The inclusion criteria were patients aged 18 and older with primary or secondary osteoarthritis, with a functionally intact rotator cuff and glenoid morphology of Walch type A1, A2, or B1. Randomization to either TSA or HA occurred intraoperatively after full surgical preparation for glenoid prosthetic implantation in all patients. The primary outcome measure was the Constant–Murley scores (CMS) at 5-year follow-up. Secondary outcomes were patient-reported outcomes (Shoulder Pain and Disability Index [SPADI], EQ-5D, and EQ VAS), adverse events, and implant survivorship at 5-year follow-up.

**Results:**

There were 79 patients eligible, of whom 40 and 39 patients were randomized to receive TSA or HA, respectively. The dropout rate at 5 years was 34% due to 27 of 79 patients withdrawing consent or being lost to follow-up. The mean CMS improved from preoperative to 5 years’ follow-up for both TSA and HA treatment groups. At 5 years, the TSA group had a significantly better mean CMS (77, 95% confidence interval [CI] 72–82) than the HA group (65, CI 57–73). The mean treatment difference was 12 (Cl 2.8–22; P = 0.01). The adverse event rate and relative risk of revision were not significantly different between the groups.

**Conclusion:**

In patients with glenohumeral osteoarthritis randomized to either TSA or HA, TSA was the favorable approach based on less pain and better joint function 5 years after surgery.

Shoulder arthroplasty is accepted as the treatment of choice for patients with glenohumeral degenerative joint diseases and has generally provided good pain relief and functional improvement [1,2]. Current surgical treatment options for rotator cuff-intact glenohumeral osteoarthritis without excessive glenoid retroversion include anatomic total shoulder arthroplasty (TSA) and humeral hemiarthroplasty (HA).

Although TSA has been perceived as superior to HA in pain relief and functional recovery, previous randomized controlled trials (RCTs) were underpowered and not able to demonstrate functional advantages [3,4]. Because HA offers a simpler surgical technique, shorter operative time, and the potential advantage of better preservation of bone stock [3,5,6], it would be important to clearly establish the superiority of TSA. The goal of the study was to undertake intraoperative randomization, after the necessary soft tissue releases had been sufficiently performed to expose the glenoid fossa for potential glenoid surface replacement, before proceeding with either HA or TSA. This study is unique in setting out to demonstrate the difference, if any, between HA and TSA after full surgical preparation of the joint performed identically in all patients, such that soft tissue balance and its consequences are no longer a determinant in functional outcomes.

The primary objective was to evaluate whether TSA was superior to HA regarding Constant–Murley Score (CMS) 5 years after surgery. The secondary objective was to compare outcomes using the Shoulder Pain and Disability Index (SPADI), duration of surgery, implant-related adverse events (AEs), implant revision, and quality of life.

## Methods

### Study design

This study was an international, multicenter, RCT with patients followed up at 6 months, 1 year, and then annually up to 5 years. The study is reported in accordance with CONSORT guidelines. All outcome measures as registered with Clinicaltrials.gov (NCT01288066) are reported.

### Patients

As glenoid morphology has been shown to affect outcomes [7,8], we limited the patient population to those with Walch type A1, A2, and B1 glenoid morphology. The inclusion criteria were patients older than 18 years diagnosed with primary or secondary osteoarthritis of different etiologies, a functionally intact rotator cuff, and glenoid morphologic type A1, A2, or B1 according to Walch [9], based on preoperative CT scans or radiographs. The preoperative exclusion criteria were patients with Walch type B2 or C glenoid, acute humeral fracture, infection in the shoulder under investigation, severe medically unmanaged systemic disease, or treated with a prior prosthesis. Patients who consumed substances that would preclude reliable assessment, were pregnant, prisoners, or participated in any other medical device or medicinal product study in the previous month that could influence the results of the study were also excluded. Intraoperative decision to use implants other than the Epoca System under investigation and diagnosis of total rupture of subscapularis or supraspinatus tendon were both exclusion criteria.

### Interventions

The devices under investigation were the Epoca Shoulder Arthroplasty System (Epoca Resurfacing Head [RH], Epoca Stem, and Epoca Glenoid; Synthes GmbH, Zuchwil, Switzerland) with or without glenoid resurfacing using an anatomic ultra-high-molecular-weight polyethylene (UHMWPE) glenoid component. A summary of implant components is provided in the Supplementary Table (see Appendix). The relevant implant design features imply that adverse features of previous prostheses were avoided, including non-anatomic humeral head height, offset, and metaphyseal overhang (generating asymmetric and adverse tension in the soft tissue envelope). The versatile design of the implant system is based on studies of native anatomy, with components available to provide patient-specific treatment with best fit and function [10]. Surgeries were performed according to the manufacturer’s technique guide. Treating surgeons were free to choose between a stemmed humeral component or a resurfacing head. Postoperative treatments were performed according to the standard of care at the study sites.

### Outcome measures

The primary outcome was assessed using the validated CMS score for shoulder function and pain [11-13] at 6-month, 1-year, 3-year, and 5-year follow-up. Secondary outcome measures included duration of surgery (from skin incision to end of wound closure), patient-reported outcomes at 6-month, 1-year, 3-year, and 5-year follow-up (Shoulder Pain and Disability Index [SPADI]) [14-16], quality of life measured by the EuroQol 5-dimensional 3-level (EQ-5D-3L) index and EQ Visual Analog Scale (VAS) questionnaires, and implant revision rate after 5-year follow-up via Kaplan–Meier survivorship analysis. Patients from Linköping, Sweden were not included in the SPADI scores because a validated translation of SPADI was not available in Swedish. Adverse events were documented over the entire follow-up period as defined by the ISO 14155 guideline [17].

### Hypothesis and sample size

We hypothesized that patients treated with TSA would have a higher mean CMS (with a score of 100 as best possible) than those treated with HA at 5 years after surgery. The null hypothesis (H0) was that there would be no difference in the CMS between the 2 treatments 5 years after surgery:

H0: CMS (TSA) – CMS (HA) = 0

The alternative hypothesis (H1) was that the CMS would differ between the 2 treatments 5 years after surgery:

H1: CMS (TSA) – CMS (HA) ≠ 0

Sample size calculation was performed using East Version 5.0.0 (Cytel Statistical software, Cambridge MA, USA), assuming a minimal clinically relevant difference (MCID) in CMS of 15 points (a value higher than the range given in the current shoulder arthroplasty literature [18-24]), a standard deviation (SD) of 20 points 5 years after surgery, significance level of 5%, power of 80%, and equal treatment groups. This resulted in a required sample size of 56 patients (28 per group). Assuming a maximum of 40% patients would be lost to follow-up at 5 years, the required sample size was increased to 94 patients (47 in each treatment group).

Due to slow patient recruitment, the enrollment was terminated before reaching the planned 94 patients. Instead, focus was placed on a lower dropout rate than was used for sample size calculation at 5 years to provide sufficient statistical power.

### Randomization and blinding

Randomization was planned at a central location using a block randomization method and stratification for each participating center: 2 blocks of irregular sizes and random order to ensure unbiased treatment allocation were generated for each study site using the “ralloc” command from the Stata software (Intercooled Stata Version 11, StataCorp LLCP, College Station, TX, USA).

After complete extrinsic and intrinsic soft tissue releases and exposure of the glenoid in all patients, an intraoperative assessment was conducted to confirm eligibility, and the randomization envelope was opened and the patient treated according to the allocation. Neither surgeons nor patients were blinded; the treating physicians also performed the follow-up examinations and outcome assessment; therefore, the outcome assessors were also not blinded.

### Statistics

Statistical analyses were conducted according to the intention-to-treat principle on all patients enrolled and randomized, i.e., the full analysis population. Due to the randomized design, the patient groups were not expected to differ in their baseline characteristics and no statistical tests were performed.

The outcomes of the CMS, SPADI, EQ-5D index, and EQ VAS were summarized using descriptive statistics of mean, SD, median, and interquartile range (IQR).

The primary efficacy analysis was conducted using a multivariable linear regression model with heteroscedasticity-consistent standard errors of multiply imputed data, adjusted for the potential prognostic factors, i.e., preoperative CMS, baseline horizontal glenoid morphology according to Walch as assessed on preoperative images, and study center (the Innsbruck site had fewer than 5 patients and was pooled with the Freiburg site based on geographical location). Scores and treatment differences were given using least squares means. Missing data for the primary efficacy analysis (i.e., the CMS) were imputed first according to a Markov Chain Monte Carlo method followed by the sequential regression method using SAS PROC MI option MONOTONE REG (https://www.sas.com/en_gb/home.html) to obtain 100 fully imputed data sets. The imputation model included treatment group, study center, horizontal glenoid morphology, and the CMS at baseline as well as at all available follow-up visits. 4 sensitivity analyses were performed to test the robustness of the results using the following approaches: (i) complete-case analysis using Welch’s t-test that included only patients with no missing data, (ii) complete-case analysis with multivariable linear regression under the same conditions as the primary efficacy analysis, (iii) last observation carried forward (LOCF) analysis (i.e., substituting the last available outcome for missing 5-year CMS) with multivariable linear regression, (iv) LOCF analysis with nonparametric randomization-based analysis of covariance, and (v) mixed-effects models for repeated measures (MMRM).

Comparison of SPADI, EQ-5D index, and EQ VAS scores at 5 years between treatment groups was conducted using multivariable linear regression models with heteroscedasticity-consistent standard errors, adjusted for potential prognostic factors using a complete-case approach. Scores and treatment differences were given using least squares means.

As the duration of surgery was likely to be right skewed, the Wilcoxon rank sum test was used for comparing the treatments. Implant revision rates were assessed for both treatments with confidence intervals (CI) calculated using the Clopper Pearson method and the risks were compared using Fisher’s exact test at 5% significance level. Implant survivorship was assessed using Kaplan–Meier survival analysis and compared using the log rank test.

### Ethics, data sharing plan, funding, and disclosures

This clinical investigation was conducted in accordance with the ethical principles of the Declaration of Helsinki and its amendments, the International Council for Harmonisation Good Clinical Practice (ICH GCP) guidelines, the European Standard EN ISO14155/2003-2011, and local laws and regulations where the research was conducted. Ethics approval was obtained from: University of Freiburg, number 83/11/01 (July 21, 2011); Hannover Medical University, number 6065 (February 08, 2012); Innsbruck Medical University, number UN4467 (February 09, 2011); and Linköping University, number 2012/12-31 (March 26, 2012).

Potentially eligible patients were approached by the study personnel, who explained the purpose, procedures, and risk and benefits of the study, alternatives to participation, and data protection. Patients choosing to participate would then sign and date an informed consent form before enrollment.

Individual researchers may contact the corresponding author for access to the original, aggregated, and anonymized datasets for research purposes.

The current study was funded by the AO Foundation via the AO Technical Commission Trauma network. Declared conflicts of interest are as follows: SL is a lecturer for J&J MedTech, a member of AO, the OSApp advisory board, and the editorial college of AO Surgery Reference, and the chair of the AO UEGEG. MJ is an advisor and lecturer for J&J MedTech, a member of AO, the OSApp advisory board, author for AO Surgery Reference, a member of the AO Sports curriculum development committee, and a former member of AO UEGEG. TB received funding from J&J MedTech for lectures, patient forums, course participation, travel expenses, and organization of observerships. AJ is an employee of AO Foundation. The remaining authors declare no conflict of interest. Complete disclosure of interest forms according to ICMJE are available on the article page, doi: 10.2340/17453674.2025.44946

## Results

### Patient characteristics

**Figure 1:**
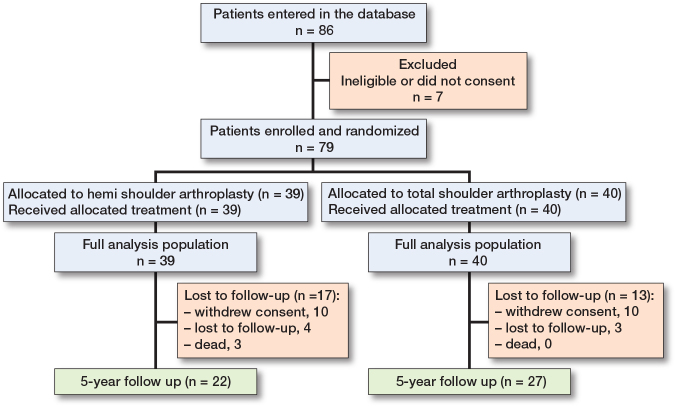
Patient flowchart.

Between January 2012 and July 2016, 86 patients were recruited from 4 centers in Germany, Sweden, and Austria. The last follow-up visit was in May 2021. Of the 86 patients entered in the database, 7 were ineligible and excluded from the study based on the defined exclusion/inclusion criteria. The 79 eligible patients included in the analysis were randomized to receive HA (n = 39) or TSA (n = 40) (Figure 1). The dropout rate at 5 years was 27 of 79 patients (34%) plus 3 deaths, i.e., 30 randomized patients did not complete the study (Figure 1). In the HA group, 10 patients withdrew consent, and 4 patients were lost to follow-up, while there were 10 patients and 3 patients, respectively, who dropped out in the TSA group. However, all 79 eligible patients were included in the primary analysis following an intention-to-treat principle. All 3 deaths during the study period occurred in the HA group. The causes for the deaths were heart failure, acute vascular obliteration of leg, and pancreatic cancer.

Baseline characteristics showed that the patient population had a median age of 69 years (IQR 60–73). In the HA group, 23 patients were female and 16 were male, while in the TSA group, 30 were female and 10 were male (Table 1). Of the 39 HA patients, 33 were diagnosed with primary osteoarthritis and 6 with secondary osteoarthritis (3 posttraumatic and 3 from rheumatoid arthritis). Of the 40 TSA patients, 36 patients had primary osteoarthritis and 4 had secondary that developed from rheumatoid arthritis. Preoperatively, 20 patients (51%) in the HA group had Walch A1 type glenoid morphology, while in the TSA group, there was a near even distribution of glenoid morphology types. Intraoperative assessment (Table 2) of the glenoid morphology agreed largely with the preoperative assessment.

**Table 1:** Baseline characteristics and shoulder pathology of full analysis population. Values are count unless otherwise specified

Variable	HA group n = 39	TSA group n = 40
Sex (female / male)	23 / 16	30 / 10
Age, median (IQR)	68 (57–72)	70 (61–75)
BMI, median (IQR)	27.9 (24.7–30.5)	29.4 (26.3–33.4)
< 18.5	0	0
18.5 to < 25.0	12	8
25.0 to < 30.0	16	15
≥ 30.0	11	17
Smoker (no / yes)	33 / 6	35 / 5
Indications		
Primary osteoarthritis	33	37
Secondary osteoarthritis		
Posttraumatic	3	4
Rheumatoid arthritis	3	0
Horizontal glenoid morphology according to Walch		
Walch type A1	20	12
Walch type A2	6	15
Walch type B1	13	13
Previous surgeries on the shoulder under investigation		
No / Yes	28 / 11	34 / 6
Capsular pattern **^a^**		
Positive capsular pattern	38	35
Internal rotation at 90°	0	3
External rotation at 90°	1	2
Preoperative Constant–Murley scores, mean (SD)		
Pain	1.7 (2.6)	2.5 (2.8)
Activities of daily living	7.8 (3.2)	8.8 (3.4)
Range of motion	11 (6.8)	11 (8.0)
Strength	2.8 (5.4)	1.7 (4.6)
Total score	24 (14)	24 (15)
Preoperative patient-reported outcomes, mean (SD)		
SPADI—Pain	78 (20)	74(15)
SPADI—Disability	68 (20)	66 (22)
Total SPADI	73 (18)	70 (17)
EQ-5D index	0.59 (0.29)	0.62 (0.26)
EQ VAS	64 (21)	64 (18)

BMI: body mass index, HA: hemiarthroplasty, IQR: interquartile range, SD: standard deviation, SPADI: Shoulder Pain and Disability Index, TSA: total shoulder arthroplasty.

a Capsular pattern was defined as:

1. Positive: the attribute of a limitation of ROM in any direction not otherwise explained by a change in shape or orientation of the humeral head or glenoid,

2. Internal rotation at 90°: the loss of range of internal rotation at the shoulder with the arm assisted at 90° of abduction in the scapular plane and without scapular motion (implies a contracture of the posterior capsule and translation of the humeral head in a forwards direction).

3. External rotation at 90°: the loss of range of external rotation at the shoulder with the arm assisted at 90° of abduction in the scapular plane and without scapular motion (implies a contracture of the anterior capsule and translation of the humeral head in a backwards direction).

**Table 2:** Intraoperative surgical details and soft tissue status. Values are count unless otherwise specified

Variable	HA group n = 39	TSA group n = 40
Surgery duration (skin incision to closure), minutes		
mean (SD)	101 (38)	114 (31)
median (IQR)	96 (67–125)	110 (95–128)
Fluoroscopy time, seconds		
mean (SD)	87 (334)	66 (259)
median (IQR)	8 (0–17)	11 (0–17)
Capsular release, complete	39	40
Intraoperative assessment of Walch horizontal glenoid morphology		
Walch type A1	21	16
Walch type A2	5	13
Walch type B1	13	11
Intraoperative status, supraspinatus tendon		
Intact	34	33
Partial rupture	5	7
Intraoperative status, subscapularis tendon		
Intact	37	39
Partial rupture	2	1
Total rupture of long head biceps tendon		
No	36	37
Yes	3	3

For abbreviations, see Table 1.

### Surgery

Surgical duration and fluoroscopy time showed no statistical significance in surgical duration between the HA (101 minutes) and TSA (114 minutes) groups (P = 0.08) (Table 2). Fluoroscopy was used intraoperatively in many cases to confirm the anatomic fit, security of implantation, and orientation of the components. Intraoperative assessment discerned that 67 patients (85%) had an intact supraspinatus tendon, 76 patients (96%) had an intact subscapularis tendon, and 6 patients (7.6%) had total rupture of the long head biceps tendon in the affected shoulder (Table 2).

### Primary outcome

The CMS subscale and total scores at 6-month, 1-year, 3-year, and 5-year follow-up are summarized in Table 3. The difference between comparable preoperative baseline CMS total scores (Table 1) and respective scores at 6 months’ follow-up was greater in the TSA group than the HA group. Primary efficacy analysis (Table 4) showed that, 5 years after the surgery, the mean CMS in the TSA group was 77 (CI 72–82), higher than in the HA group (65, CI 57–73); P = 0.01). The between-group treatment difference was 12 (CI 2.8–22) points. In agreement with the primary analysis, all 5 sensitivity analyses showed the TSA group had better results than the HA group at 5 years (Table 4). An MMRM sensitivity analysis was performed additionally for timepoints across the entire follow-up period. There were better MMRM results for the TSA group with between-group treatment differences of 9.4 (CI 1.3–18), P = 0.02 at 6 months; 5.3 (CI –3.7 to 14), P = 0.2 at 1 year; 8.8 (CI 0.9–17), P = 0.03 at 3 years; and 12 (CI 1.4–23), P = 0.03 at 5 years, although the difference at 1 year was not statistically different.

**Table 3:** Primary outcome: Constant–Murley Scores (CMS) over the postoperative follow-up period

Variable	HA group, n = 39				TSA group, n = 40			
	6 months	1 year	3 years	5 years	6 months	1 year	3 years	5 years
Pain, n	37	32	31	22	40	37	31	26
mean (SD)	10 (4.3)	11 (5.0)	12 (3.8)	11 (5.6)	13 (2.4)	13 (3.8)	14 (2.5)	14 (1.6)
median (IQR)	10 (10–15)	10 (10–15)	15 (10–15)	15 (5–15)	15 (10–15)	15 (10–15)	15 (15–15)	15 (15–15)
Activities of daily living, n	37	32	31	22	40	37	31	26
mean (SD)	16 (4.5)	17 (4.6)	17 (4.7)	16 (5.2)	18 (3.2)	18 (4.3)	19 (2.3)	19 (1.8)
median (IQR)	18 (12–20)	20 (14–20)	20 (17–20)	20 (12–20)	20 (18–20)	20 (18–20)	20 (19–20)	20 (20–20)
Range of motion, n	37	32	31	22	39	36	31	25
mean (SD)	26(8.6)	28 (8.1)	30 (8.2)	29 (9.3)	29 (7.4)	31 (7.4)	34 (4.9)	33 (4.7)
median (IQR)	26 (20–32)	29 (23–35)	32 (26–36)	34 (22–36)	30 (24–36)	32 (26–36)	36 (32–38)	34 (30–36)
Strength **^a^**, n	37	32	31	22	40	37	31	26
mean (SD)	9.1 (5.6)	9.7 (7.2)	11 (5.9)	9.5 (6.4)	9.5 (6.5)	10 (6.5)	13 (7.4)	11 (5.9)
median (IQR)	8.8 (5.5–12)	9.0 (5.5–14)	8.8 (6.6–14)	8.8 (5.5–11)	8.3 (4.4–15)	8.8 (6.6–14)	9.9 (6.8–20)	9.9 (7.1–15)
Total CMS, n	37	32	31	22	39	36	31	25
mean (SD)	61 (19)	66 (22)	70 (18)	66 (23)	70 (16)	72 (16)	80 (12)	78 (9.5)
median (IQR)	65(51–74)	71 (49–82)	75 (61–81)	76 (42–82)	74(61–82)	74 (65–82)	80 (72–90)	79 (73–84)

For abbreviations, see Table 1.

a The investigators agreed that as any strength at any position could be of value to the patient (e.g., strength at ranges below 90° of abduction remains a useful attribute for the majority of daily activities), a pragmatic view was taken toward testing and reporting strength: the testing was performed in the same way pre- and postoperatively so the observer bias would be the same for study center cohorts. The aim was to achieve strength-testing at as close to 90° of abduction as painlessly possible using a standardized and agreed protocol. The overall bias was in favor of a higher CMS for both pre- and postoperative tests, so the magnitude of the relative difference was in the same direction.

**Table 4:** Primary and sensitivity statistical analysis, comparison of Constant–Murley Scores (CMS) at 5 years

Item	HA group, n = 39		TSA group, n = 40		Difference (CI)	P value
	n	CMS mean (CI)	n	CMS mean (CI)		
Primary efficacy test a using multiply imputed data						
	39	65 (57–73)	40	77 (72–82)	12 (2.8–22)	0.01
Sensitivity test 1: Complete-case, Welch’s t-test						
	22	66 (56–76)	25	78 (74–82)	12 (1.5–23)	0.03
Sensitivity test 2: Complete-case, multivariable linear regression **^a^**						
	22	66 (55–77)	25	79 (74–83)	13 (0.7–25)	0.04
Sensitivity test 3: Last observation carried forward, multivariable linear regression **^a^**						
	36	65 (57–74)	40	77 (72–81)	11 (2.0–21)	0.02
Sensitivity test 4: Last observation carried forward, nonparametric analysis of covariance						
	36	N/A	40	N/A	12 (3.4–20)	0.01
Sensitivity test 5: Mixed-effects model						
	22	66 (60–77)	25	79 (75–82)	12 (1.4–23)	0.03

CI: 95% confidence interval, HA: hemiarthroplasty, TSA: total shoulder arthroplasty. N/A: not applicable (comparison conducted using a nonparametric model, where the least squares (LS) means were not calculated for individual groups).

a Multivariable linear regression model adjusted for preoperative CMS, baseline horizontal glenoid morphology according to Walch (as assessed on images), and study sites. Presented values are LS means and difference in LS means (using observed margins).

### Secondary outcomes

#### PROMs

Regarding the effects on patient-reported disability and quality of life, the total and subscale scores for SPADI, EQ-5D index, and EQ VAS at 6-month, 1-year, 3-year, and 5-year follow-up according to treatments are summarized in Table 5. The differences between comparable preoperative baseline SPADI total and EQ VAS scores (Table 1) and respective scores at 6-month follow-up were greater in the TSA group than in the HA group. Visual inspection of the numbers showed that over the follow-up period the TSA group had consistently lower disability than the HA group, in both the SPADI total score and the subscale scores, with the median scores at 5 years being 8.3 (IQR 0.0–40) points in the HA group and 3.1 (IQR 0.0–9.9) in the TSA group (Table 5). Multivariable linear regression analysis of SPADI scores using complete cases showed a mean difference of –16 (CI –30 to –2.1; P = 0.03) at 5 years, favoring the TSA group (Table 6).

**Table 5:** Patient-reported outcomes: disability and quality of life scores over the postoperative follow-up period

Variable	HA group, n = 39				TSA group, n = 40			
	6 months	1 year	3 years	5 years	6 months	1 year	3 years	5 years
SPADI—Pain, n	31	24	25	17	34	29	25	20
mean (SD)	35 (26)	30 (29)	28 (32)	20 (27)	19 (18)	13 (23)	9.7 (15)	3.7 (5.6)
median (IQR)	40 (10–56)	24 (5–47)	14 (2–48)	4 (0–44)	14 (4–28)	2 (0–14)	2 (0–18)	0 (0–7)
SPADI—Disability, n	31	26	25	17	34	31	25	20
mean (SD)	31 (21)	25 (24)	20 (30)	17 (25)	20 (23)	16 (23)	6.9 (12)	6.1 (7.9)
median (IQR)	31 (13–48)	22 (3.8–36)	3.8 (0–31)	2.5 (0–36)	11 (1.3–35)	3.8 (0–21)	0 (0–10)	1.3 (0–12)
Total SPADI, n	31	24	25	17	34	29	25	20
mean (SD)	33 (22)	26 (26)	24 (30)	19 (26)	20 (19)	14 (23)	8.3 (12)	4.9 (5.5)
median (IQR)	29 (16–45)	23 (4.6–39)	8.0 (2.6–40)	8.3 (0–40)	13 (3.5–37)	3.1 (0–16)	2.6 (0–15)	3.1 (0–9.9)
EQ-5D index, n	37	32	30	22	39	37	31	26
mean (SD)	0.86 (0.20)	0.84 (0.21)	0.78 (0.29)	0.83 (0.24)	0.89 (0.14)	0.86 (0.19)	0.94 (0.09)	0.90 (0.17)
median (IQR)	0.87 (0.87–1)	0.87 (0.84–1)	0.87 (0.70–1)	0.87 (0.79–1)	0.87 (0.80–1)	0.87 (0.79–1)	1.00 (0.87–1)	1.00 (0.78–1)
EQ VAS, n	37	32	31	22	40	35	31	26
mean (SD)	72 (18)	72 (24)	68 (24)	73 (21)	77 (15)	72 (23)	83 (12)	76 (23)
median (IQR)	80 (60–85)	80 (62–90)	70 (50–90)	80 (60–90)	80 (68–90)	75 (60–90)	80 (75–94)	80 (70–90)

For abbreviations, see Table 1.

The Shoulder Pain and Disability Index (SPADI) subscales and total score range from 0 to 100 with higher scores indicating greater impairment.

**Table 6:** Multivariable linear regression analysis a: comparison of disability and quality of life at 5 years

Item	HA group, n = 39		TSA group, n = 40		Difference (CI)	P value
	n	mean (CI)	n	mean (CI)		
SPADI	16	20 (8.4–32)	20	4.3 (0–10)	–16 (–30 to –2.1)	0.03
EQ-5D index	20	0.82 (0.70–0.95)	26	0.90 (0.80–1.0)	0.08 (–0.10 to 0.26)	0.4
EQ VAS	20	72 (60–83)	26	75 (65–86)	3.6 (–12 to 20)	0.7

For abbreviations, see Table 4.

a Multivariable linear regression analysis with heteroscedasticity-consistent standard errors of complete cases, adjusted for preoperative outcome scores, baseline Walch glenoid morphologies, and study sites. Presented values are least squares (LS) means and difference of LS means (using observed margins).

Both EQ-5D index and EQ VAS scores plateaued starting at 6 months after the surgery in both treatment groups (see Table 5); multivariable linear regression analyses showed no difference between the treatments at 5 years (Table 6).

There was no difference in median surgical duration between the HA group (96, IQR 67–125 minutes) and TSA group (110, IQR 95–128 minutes; P = 0.08).

### Adverse events

At the 5-year follow-up, 20 patients in the TSA group and 24 patients in the HA group had at least 1 AE, resulting in a relative risk ratio of 0.79 (CI 0.49–1.3; P = 0.4) (Table 7). 1 patient in each group had loosening of the humeral stem. There was no glenoid migration or loosening of glenoid in the TSA group. There were no reported cases of the following AEs: intraoperative humerus fracture, intraoperative glenoid perforation or fracture, migration of stem, loosening of Epoca RH, migration of Epoca RH, periprosthetic infection, material wear, loosening of glenoid (one reported case in the HA group but only after a revision from HA to TSA), migration of glenoid, late humerus fracture, humeral head dislocation, decentralization, joint stiffness, and axillary nerve palsy.

**Table 7:** Summary of reported adverse events

Adverse events ^a^	HA n = 39	TSA n = 40	Relative risk ^b^ (CI ^c^)	P value ^d^
Any adverse event	24	20	0.79 (0.49–1.3)	0.4
Anticipated adverse event				
Loosening of humeral stem	1	1	0.99 (0.24–4.0)	1.0
Humeral head subluxation	1	0	0.49 (0.39–0.61)	0.5
Secondary glenoid erosion	7	N/A	N/A	N/A
Arthrofibrosis	1	0	0.49 (0.39–0.61)	0.5
Rotator cuff tear	1	4	2.8 (0.44–15)	0.4
Hematoma requiring intervention	1	0	0.49 (0.39–0.61)	0.5
Unexplained pain	1	0	0.49 (0.39–0.61)	0.5
Superficial wound infection	1	0	0.49 (0.39–0.61)	0.5
Heterotopic bone formation	4	2	0.72 (0.39–1.3)	0.4
Other event	21	16	0.76 (0.48–1.2)	0.3

For abbreviations, see Table 4.

a Number of patients with at least 1 adverse event; 1 patient could be presented in multiple cells.

b Relative risk comparing total arthroplasty with hemiarthroplasty.

c 95% CI calculated using the Clopper Pearson method.

d Fisher’s exact test.

Over the study period, 6 HA patients had 6 primary implant revisions to a TSA (3 anatomical, 2 reverse, and 1 unknown TSA), with 1 patient having a second TSA from anatomical to reverse. 2 TSA patients had 2 primary implant revisions (1 anatomical, 1 reverse). The cause of revision was pain in all cases, except for 1 unknown reason in the HA group and 1 case of septic loosening of the humeral component in the TSA group.

**Figure 2:**
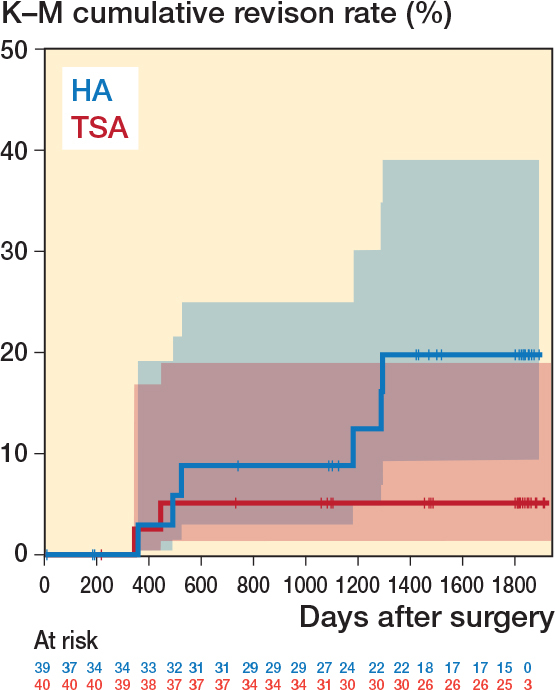
Kaplan–Meier survivorship curve of time to first implant revision by treatment group. At 5 years (~1,826 days), the implant revision rate was 19.8% (95% confidence interval [CI] 9.3–39) in the hemiarthroplasty (HA) group and 5.1% (CI 1.3–19) in the total shoulder arthroplasty (TSA) group.

The estimated risk of at least 1 implant revision at 5 years was 15% (CI 5.9–31) in the HA group and 5.0% (CI 0.6–17) in the TSA group. The relative risk of having an implant revision was 0.33 (CI 0.07–1.5) for TSA compared with HA, which was not statistically significant (P = 0.2). Kaplan–Meier analysis determined that at 5 years the implant revision rate was 20% (CI 9.3–39) in the HA group and 5.1% (CI 1.3–19) in the TSA group (log-rank P = 0.1; Figure 2).

## Discussion

We aimed to investigate whether patients treated with TSA compared with HA had a superior outcome 5 years after surgery. We showed a between-group treatment difference of 12 points (CI 2.8–22) for CMS, favoring TSA (P = 0.01). At the time of establishing the study protocol, the only CMS MCID validated was from 1 cohort study on rotator cuff repair, which reported a difference of 10.4 [18]. While the CMS MCID was then set in the Epoca protocol at 15 points, current research has consolidated reported MCIDs from the last decade [19-24], even specifically for anatomical TSA [21,24]. A 2025 systematic review reported a CMS MCID of mean 10.3 (range 5.7–12.8) for anatomical TSA [24], with an MCID of 5.7 from a cohort study by Simovitch et al. 2018 [19]. Therefore, while the literature suggests variability in the MCID thresholds [20,24], the difference between TSA and HA observed in the current study is in alignment with the reported benchmarks in the literature.

Secondary outcome showed less disability in the TSA group at 5 years, with a between-group treatment difference of –16 (CI –30 to –2.1) points. An MCID for SPADI was not set as a benchmark for this study, but the 2025 systematic review reports a comparable range of 14.6 to 21.6 for the MCID for anatomical TSA [24].

Although TSA has been perceived as superior to HA in pain relief and functional recovery, HA offers a simpler surgical technique, shorter operative time, and potential advantage of better preservation of bone stock [3,5,6]. The shorter operating time reflects the lack of need to expose the glenoid fossa sufficiently for instrumentation. The resulting asymmetric capsular tensions in HA may result in progressive glenoid erosion requiring secondary surgery [25]. An initial concern of TSA was its technical complexity and longer operative time [3,6]. In our study, complete capsular release was applied to all patients and the surgical duration was 101 minutes (HA) and 114 minutes (TSA); the lack of significance (P = 0.08) of this difference of 13 minutes is important: all patients in the study had an equal focus on adequate capsular releases, sufficient to gain exposure of the glenoid as if a glenoid surface replacement was to be implanted. This contrasts with another study comparing HA with TSA in which the difference in operating time exceeded 30 minutes, suggesting that the soft tissue preparation in the HA group was less extensive than for the TSA group, thus confounding the comparison between the groups [3]. Soft tissue releases are essential for a successful glenoid surface implantation in TSA and create an optimal soft tissue balance for better motion. While glenoid surface replacement seems to deliver optimal pain relief and range of motion, the attendant risk of AEs related to the glenoid component (e.g., wear, loosening) leading to revision surgery remains of concern [26,27]. A matched HA versus TSA cohort study published in 2024 analyzed real-world data from the UK National Joint Registry on adverse outcomes in 11,556 patients with glenohumeral osteoarthritis and an intact rotator cuff. The study concluded that the risk of revision was higher at 8 years following HA compared with TSA, particularly for patients aged 60 years and younger. Rotator cuff insufficiency was the most common reason for revision [28]. In our study we did not see any statistically significant difference regarding the risk of revision at 5 years between HA and TSA, and the majority of revisions in both groups were due to persistent pain.

A guideline from the American Academy of Orthopedic Surgeons gave a strong recommendation for using TSA over HA [29,30]; the “strong recommendation” rating was based mainly on better clinical and patient-reported outcomes reported in 2 high-quality RCTs and a meta-analysis of studies with various levels of evidence [30]. It is worthwhile noting that a closer look into the 2 RCTs, both with relatively short follow-up (mean follow-up of 35 months and 2 years) [3,4], revealed that both were underpowered. Except for the better CMS pain score (and internal rotation) after TSA, the functional subscale scores and quality of life scores showed no difference between HA and TSA [3,4] but a major benefit of the TSA was pain relief [31,32].

Compared with previous studies, the current study employing intraoperative randomization is the only RCT that clearly demonstrated the benefits of TSA over HA in both pain and function 5 years after surgery. Although our enrolled number of patients did not reach the target sample size, the dropout rate was lower than expected and we could demonstrate that the TSA group had better total and subscale scores at 5 years in both the CMS and SPADI, as well at most of the scheduled visits. With this, the robustness of the results from the primary analysis using an intention-to-treat approach was further supported by multiple sensitivity analyses of the CMS at 5 years following both complete-case and last-observation-carried-forward approaches.

In alignment with our results, a meta-analysis concluded that there was an 8% relative increase in the postoperative patient-reported outcomes in the TSA cohort compared with that in HA. In addition, the HA cohort had a significant 2-fold increase in the combined revision and complication rates compared with TSA [33]. The results from our RCT enhance the findings of this meta-analysis.

Regarding AE, we showed no glenoid component loosening or migration, but longer follow-up will be necessary to determine the survivorship of the TSA.

### Strengths and limitations [3,4,32]

The strength of the current study is its larger sample size and mid-term (5 years) follow-up. In addition, the current study assessed and confirmed intraoperatively the glenoid morphology and the state of the rotator cuff—2 factors that may influence the outcome of TSA [34,35], which increased the reliability of the results.

The limitation of the current study is that it is not powered to study a difference in revision rate, which also needs a longer follow-up time.

### Conclusion

In patients with glenohumeral osteoarthritis randomized to either TSA or HA, TSA was the favorable approach based on less pain and better joint function 5 years after surgery.

*In perspective*, we have demonstrated that even if optimal soft tissue preparation is undertaken in the case of an HA for osteoarthritis of the shoulder, the short- and medium-term clinical and patient-related outcomes are inferior to those of a TSA. Protection of the glenoid with a surface replacement, i.e., a TSA, appears important for optimal recovery of comfort and function.

## Notes

SL originated the study idea. SL, NS, MJ, LA, TB, MB, and AJ designed the study, collected the data, approved the manuscript, analyzed and interpreted the results, and agreed to be accountable for the work.

The authors thank the AO Foundation Innovation Translation Center Clinical Evidence team for conducting the study, performing the statistical analysis, and manuscript writing. The authors thank Greta Thompson-Steckel of the AO Innovation Translation Center for providing medical writing support in accordance with Good Publication Practice (GPP 2022) guidelines.

Handling co-editors: Jeppe Vejlgaard Rasmussen and Robin Christensen

*Acta* thanks Anne Karelse and Björn Salomonsson for help with peer review of this manuscript.

## Appendix

**Supplementary Table:** Choice of humeral and glenoid components

Item	HA group n = 39	TSA group n = 40	Total n = 79
Type of humeral component, n (%)			
Stemmed	22	21	43 (54)
Resurfacing head	17	19	36 (46)
Reason for choice of humeral component, n (%)			
Age of patient	21	23	44 (56)
General constitution of patient	10	8	18 (23)
Problems with visibility of glenoid leading to use of stemmed implant	0	1	1 (1.3)
Surgeon’s preference	29	35	64 (81)
Technical reasons	2	3	5 (6.3)
Other reasons	3	2	5 (6.3)
Type of glenoid component, n (%)			
All-polyethylene glenoid (cemented)	N/A	17	17 (43)
Hybrid glenoid with shell screws (cemented)	N/A	4	4 (10)
Pressfit metalback glenoid (cementless, no screws)	N/A	19	19 (48)

HA: hemiarthroplasty, N/A: not applicable, TSA: total shoulder arthroplasty.
